# Supplementary material for: The interactome of intact mitochondria by cross-linking mass spectrometry provides evidence for coexisting respiratory supercomplexes
Source: Mol Cell Proteomics. 2017 Dec 8;17(2):216–32. doi: 10.1074/mcp.RA117.000470 (PMC5795388; doi:10.1074/mcp.RA117.000470)
Supplement: Supplemental Data [file supp_RA117.000470_133922_0_supp_23978_fzffwf.zip › spectra_annotation/mito_DR_spectra_annotation/150-1-15-1-16-1.pdf]

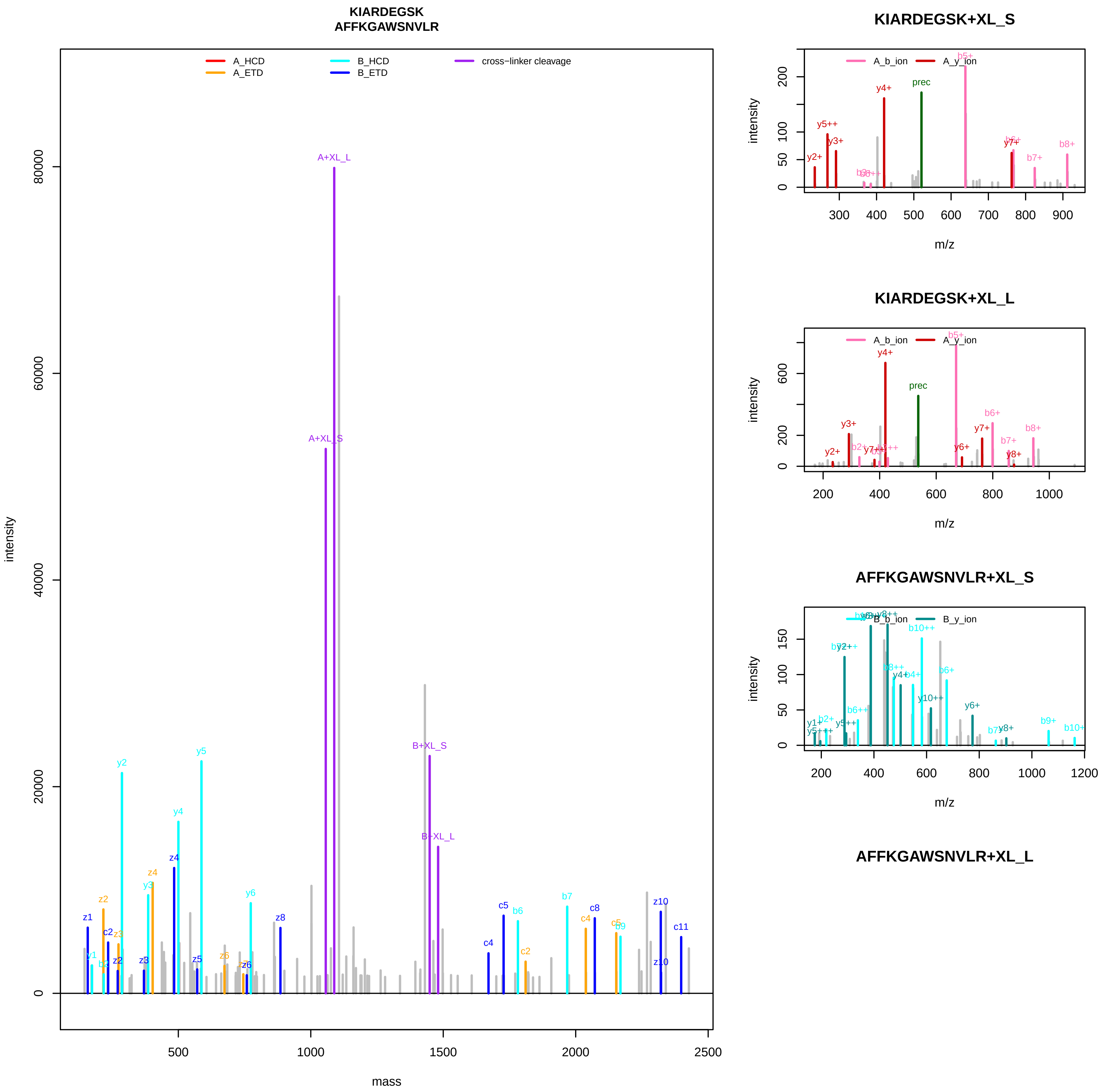
KIARDEGSK
AFFKGAWSNVLR
A_HCD
A_ETD
B_HCD
B_ETD
cross−linker cleavage
A+XL_L
A+XL_S
B+XL_S
B+XL_L
z1
y1
b2
c2
z2
z3
y2
z3
y3
z4
z4
y4
z5
y5
z6
z6
y6
z8
c4
c5
b6
c2
b7
c4
c8
c5
b9
z10
z10
c11
intensity
mass
0
20000
40000
60000
80000
500
1000
1500
2000
2500
KIARDEGSK+XL_S
A_b_ion
A_y_ion
y2+
y5++
y3+
y4+
prec
b5+
y7+
b6+
b7+
b8+
intensity
m/z
0
50
100
200
300
400
500
600
700
800
900
KIARDEGSK+XL_L
A_b_ion
A_y_ion
y2+
y3+
y4+
prec
b5+
y6+
b6+
y7+
b7+
y8+
b8+
intensity
m/z
0
200
600
200
400
600
800
1000
AFFKGAWSNVLR+XL_S
B_b_ion
B_y_ion
y1+
y5+++
b2+
y5++
b6++
b10++
y4+
b4+
b6+
y10++
y6+
b7+
y8+
b9+
b10+
intensity
m/z
0
50
100
150
200
400
600
800
1000
1200
AFFKGAWSNVLR+XL_L
